# Homologous Pairing Activities of Two Rice RAD51 Proteins, RAD51A1 and RAD51A2

**DOI:** 10.1371/journal.pone.0075451

**Published:** 2013-10-04

**Authors:** Yuichi Morozumi, Ryohei Ino, Shukuko Ikawa, Naozumi Mimida, Takeshi Shimizu, Seiichi Toki, Hiroaki Ichikawa, Takehiko Shibata, Hitoshi Kurumizaka

**Affiliations:** 1 Laboratory of Structural Biology, Graduate School of Advanced Science and Engineering, Waseda University, Shinjuku, Tokyo, Japan; 2 RIKEN, Wako, Saitama, Japan; 3 National Institute of Agrobiological Sciences, Tsukuba, Ibaraki, Japan; Saint Louis University, United States of America

## Abstract

In higher eukaryotes, RAD51 functions as an essential protein in homologous recombination and recombinational repair of DNA double strand breaks. During these processes, RAD51 catalyzes homologous pairing between single-stranded DNA and double-stranded DNA. Japonica cultivars of rice (*Oryza sativa*) encode two RAD51 proteins, RAD51A1 and RAD51A2, whereas only one RAD51 exists in yeast and mammals. However, the functional differences between RAD51A1 and RAD51A2 have not been elucidated, because their biochemical properties have not been characterized. In the present study, we purified RAD51A1 and RAD51A2, and found that RAD51A2 robustly promotes homologous pairing *in vitro*. RAD51A1 also possesses homologous-pairing activity, but it is only about 10% of the RAD51A2 activity. Both RAD51A1 and RAD51A2 bind to ssDNA and dsDNA, and their DNA binding strictly requires ATP, which modulates the polymer formation activities of RAD51A1 and RAD51A2. These findings suggest that although both RAD51A1 and RAD51A2 have the potential to catalyze homologous pairing, RAD51A2 may be the major recombinase in rice.

## Introduction

DNA double strand breaks (DSBs) are generated by DNA damaging agents, ionizing radiation, and collapsed DNA replication forks [1], [2]. Unrepaired DSBs cause chromosomal aberrations, leading to tumorigenesis or cell death. Homologous recombination is the error-free repair pathway for DSBs in mitosis [3], [4], [5], and is also required for the proper segregation of homologous chromosomes during the first meiotic cell division [6], [7].

In eukaryotes, RAD51, which is the central recombinase in homologous recombination, has been identified as a homologue of *Escherichia coli* RecA [8], [9], [10], [11]. DMC1, another RecA homologue, has also been found in eukaryotes [12], [13]. RAD51 is involved in both mitotic and meiotic homologous recombination [8], whereas DMC1 functions exclusively in meiosis [12]. During homologous recombination, RAD51 catalyzes homologous pairing, by which single-stranded DNA (ssDNA) forms new Watson-Crick base pairs (heteroduplex) with a complementary strand of homologous double-stranded DNA (dsDNA) in an ATP-dependent manner [14], [15], [16], [17], [18]. The meiosis-specific DMC1 also possesses homologous-pairing activity [19], [20], [21], [22]. In the homologous-pairing reaction, RAD51 and DMC1 first bind to ssDNA, and form helical nucleoprotein filaments. The resulting nucleoprotein filaments then bind to dsDNA, and facilitate heteroduplex formation in the ternary complex containing ssDNA, dsDNA, and either RAD51 or DMC1.

In *Saccharomyces cerevisiae*, strains bearing mutations in the *Rad51* gene are quite sensitive to DNA damage agents, but are viable [3], [8]. In mice, the *RAD51* gene knockout leads to early embryonic lethality [23], [24]. Similarly, deletion of the *RAD51* gene in chicken DT40 cells causes the accumulation of chromosomal breaks and subsequent cell death [25]. These findings indicated that RAD51 is essential in higher eukaryotes. RAD51 has also been found in higher plants [26], [27], [28], [29] and a moss [30]. In maize, RAD51 facilitates proper homologous chromosome pairing in meiosis [31], [32]. The *Arabidopsis* genome has a single *RAD51* gene [27], whereas the *Zea mays* and *Physcomitrella patens* genomes contain two genes [28], [30]. In *Arabidopsis*, the loss of RAD51 function has no impact on both the vegetative and flower development, and no abnormalities were detected in mitosis, whereas the mutant results in meiotic defects and causes sterility [33]. In contrast to *Arabidopsis*, the double knockout of two *RAD51* genes significantly affects both the vegetative and generative development in *Physcomitrella* [34]. Since the two *Physcomitrella* RAD51 proteins reportedly promote homologous pairing with similar efficiencies [35], they may redundantly function in the moss.

In cultivated rice (*Oryza sativa*), a single copy of the *RAD51* gene has been reported so far in an indica cultivar [36], while the japonica cultivar group genome contains two non-allelic *RAD51A1* and *RAD51A2* genes. Interestingly, the *O. sativa* genome also contains two *DMC1* genes, *DMC1A* and *DMC1B*. Purified DMC1A and DMC1B promoted significant homologous recombination reactions *in vitro* [37], suggesting that both DMC1A and DMC1B are *bona fide* recombinases functioning in rice meiosis. However, the recombination activities of RAD51A1 and RAD51A2 from the japonica cultivar group have not been compared so far. In the present study, we purified the japonica rice RAD51A1 and RAD51A2 proteins, and found that RAD51A2 possesses robust homologous-pairing activity, which is about 10-fold higher than that of RAD51A1.

## Materials and Methods

### Protein Purification

The DNA fragments encoding the *RAD51A1* and *RAD51A2* sequences from the japonica cultivar Nipponbare (NCBI accession nos. AB080262 and AB080264, respectively) were cloned into the *Nde*I-*Bam*HI sites of the pET-15b expression vector (Novagen). In each construct, the His_6_ tag-coding sequence was fused at the N-terminal end of the *RAD51A1*- and *RAD51A2*-coding sequences. His_6_-tagged RAD51A1 and RAD51A2 were individually expressed in the *Escherichia coli* BLR(DE3) pLysS strain, in which the *recA* gene has been deleted (Novagen). The cells producing the proteins were resuspended in buffer A [50 mM Tris-HCl (pH 8.0), 2 M NaCl, 10 mM EDTA, 5 mM 2-mercaptoethanol, and 10% glycerol], and were disrupted by sonication. The cell debris was removed by centrifugation for 20 min at 27,700 × *g*, and the supernatant was mixed gently with 3 ml of cOmplete His-Tag Purification Resin (Roche) at 4°C for 1 hr. The protein-bound beads were then packed into an Econo-column (Bio-Rad Laboratories), and were washed with 150 ml of buffer A, containing 20 mM imidazole. The proteins were eluted by a 60 ml linear gradient of imidazole from 20 to 300 mM. The peak fractions were collected, and 8 units of thrombin protease (GE Healthcare Biosciences) per mg of protein were added to remove the His_6_ tag. The samples were immediately dialyzed against a buffer containing 20 mM Tris-HCl (pH 8.0), 500 mM NaCl, 5 mM EDTA, 5 mM 2-mercaptoethanol, and 10% glycerol. After removal of the His_6_ tag, RAD51A1 and RAD51A2 were loaded onto a Superdex 200 gel filtration column (HiLoad 26/60 preparation grade; GE Healthcare Biosciences), which was previously equilibrated with buffer containing 20 mM Tris-HCl (pH 8.0), 2 M NaCl, 10 mM EDTA, 5 mM 2-mercaptoethanol, and 10% glycerol. RAD51A1 and RAD51A2 were eluted from the Superdex 200 gel filtration column, dialyzed against buffer containing 20 mM HEPES-NaOH (pH 7.5), 400 mM NaCl, 0.1 mM EDTA, 2 mM 2-mercaptoethanol, and 10% glycerol, and stored at −80°C. The DNA fragments encoding the RAD51A1(A2L2) and RAD51A2(A1L2) mutants were constructed, and were cloned into the *Nde*I-*Bam*HI sites of the pET-15b expression vector (Novagen). The RAD51A1(A2L2) and RAD51A2(A1L2) mutants were then purified by the same method as for the wild type proteins. Human RAD51 was expressed in *E. coli* cells [38], and was purified as described previously [39].

### DNA Substrates

Single-stranded φX174 viral (+) strand DNA and double-stranded φX174 replicative form I DNA were purchased from New England Biolabs, and the linear dsDNA was prepared from the φX174 replicative form I DNA by *Pst*I digestion. In the D-loop formation assay, superhelical dsDNAs were prepared by a method avoiding alkaline treatment of the cells harboring the plasmid DNA [40]. For the ssDNA substrate used in the D-loop formation assay, the following HPLC-purified DNA oligonucleotides were purchased from Nihon Gene Research Laboratory: 50-mer, 5′-ATT TCA TGC TAG ACA GAA GAA TTC TCA GTA ACT TCT TTG TGC TGT GTG TA-3′. All of the DNA concentrations are expressed in moles of nucleotides.

### The ATPase Assay

The ATPase activities of the proteins were analyzed by the release of ^32^Pi from [γ-^32^P]ATP. Rice RAD51A1 or RAD51A2 (1.5 µM) was incubated at 37°C in the presence of φX174 circular ssDNA (20 µM) or linearized φX174 dsDNA (20 µM), or in the absence of DNA, in 10 µl of reaction buffer, containing 24 mM HEPES-NaOH (pH 7.5), 80 mM NaCl, 0.02 mM EDTA, 0.4 mM 2-mercaptoethanol, 2% glycerol, 1 mM MgCl_2_, 1 mM DTT, 5 or 500 µM ATP, 5 nCi [γ-^32^P]ATP, and 0.1 mg/ml BSA. At the indicated times, the reaction was stopped by the addition of 1 µl of 0.5 M EDTA, and the products were separated by thin layer chromatography on polyethyleneimine-cellulose in a 0.5 M LiCl and 1 M formic acid solution.

### The D-loop Formation Assay

The indicated amount of rice RAD51A1, RAD51A2, or human RAD51 was incubated with the ^32^P-labeled 50-mer oligonucleotide (1 µM) in the presence or absence of CaCl_2_ (1 mM) at 37°C for 5 min, in 9 µl of reaction buffer, containing 24 mM HEPES-NaOH (pH 7.5), 80 mM NaCl, 0.02 mM EDTA, 0.4 mM 2-mercaptoethanol, 2% glycerol, 1 mM MgCl_2_, 1 mM DTT, 1 mM ATP, 0.1 mg/ml BSA, 20 mM creatine phosphate, and 75 µg/ml creatine kinase. The reactions were then initiated by the addition of 1 µl of pGsat4 superhelical dsDNA (30 µM), and were continued at 37°C for 5 min. The reactions were stopped by the addition of 0.2% SDS and 1.5 mg/ml proteinase K (Roche), and were further incubated at 37°C for 15 min. After adding 6-fold loading dye, the deproteinized reaction products were separated by 1% agarose gel electrophoresis in 1× TAE buffer (40 mM Tris-acetate (pH 8.0), and 1 mM EDTA) at 4 V/cm for 2 hrs. The gels were dried and exposed to an imaging plate. The gel images were visualized using an FLA−7000 imaging analyzer (Fujifilm). We confirmed that both RAD51A1 and RAD51A2 did not promote the D-loop formation with a heterologous dsDNA (pB5Sarray, which contained 11 repeats of a sea urchin 5S rRNA gene (207-bp fragment) within the pBlueScript II SK(+) vector) under the conditions employed in the present study.

### The DNA-binding Assay

The φX174 circular ssDNA (20 µM) or the linearized φX174 dsDNA (20 µM) was mixed with rice RAD51A1, RAD51A2, or human RAD51 in the presence or absence of 1 mM ATP, in 10 µl of a standard reaction solution, containing 30 mM HEPES-NaOH (pH 7.5), 200 mM NaCl, 0.05 mM EDTA, 1 mM 2-mercaptoethanol, 5% glycerol, 1 mM MgCl_2_, 1 mM DTT, and 0.1 mg/ml BSA. The reaction mixtures were incubated at 37°C for 10 min, and were then separated by 0.8% agarose gel electrophoresis in 1× TAE buffer at 3.3 V/cm for 2 hrs. The bands were visualized by ethidium bromide staining.

### Electron Microscopy

Rice RAD51A1 or RAD51A2 (0.5 µM) was mixed with the φX174 dsDNA (0.5 µM) in 20 µl of reaction buffer, containing 30 mM HEPES-NaOH (pH 7.5), 200 mM NaCl, 0.05 mM EDTA, 1 mM 2-mercaptoethanol, 5% glycerol, 1 mM MgCl_2_, and 1 mM ATP, and was incubated at 37°C for 10 min. Samples (3 µl) were adsorbed on a carbon grid and stained with 2% uranium acetate. The samples were examined with a JEM2000FX electron microscope (JOEL).

### Gel Filtration Analysis

Rice RAD51A1 or RAD51A2 (70 µg) was incubated with or without ATP (1 mM) or ADP (1 mM) at room temperature for 30 min, in a reaction mixture containing 20 mM HEPES-NaOH (pH 7.5), 355 mM NaCl, 0.1 mM EDTA, 2 mM 2-mercaptoethanol, 10% glycerol, and 1 mM MgCl_2_. The samples were then analyzed by Superdex 200 GL 10/300 (GE Healthcare BioSciences) gel filtration chromatography. The elution buffer contained 20 mM HEPES-NaOH (pH 7.5), 400 mM NaCl, 0.1 mM EDTA, 2 mM 2-mercaptoethanol, and 1 mM MgCl_2_, and the elution profiles of RAD51A1 or RAD51A2 were monitored by UV absorption at 280 nm.

### The pull-down Assay with Ni-NTA Beads

Purified His_6_-tagged RAD51A1 (2.0 µg) was mixed with RAD51A1 (4.0 µg) or RAD51A2 (4.0 µg) in 60 µl of binding buffer, containing 19 mM HEPES-NaOH (pH 7.5), 380 mM NaCl, 0.095 mM EDTA, 1.9 mM 2-mercaptoethanol, 0.025% Triton X-100, and 9.5% glycerol, and then Ni-NTA agarose beads (1.5 µl, 50% slurry) were added. After an incubation at room temperature for 1 hr, the beads were washed two times with 300 µl of wash buffer, containing 20 mM HEPES-NaOH (pH 7.5), 400 mM NaCl, 0.1 mM EDTA, 2 mM 2-mercaptoethanol, and 10% glycerol. The proteins bound to the beads were fractionated by 10% SDS-PAGE, and the bands were visualized by Coomassie Brilliant Blue staining.

## Results

### Purification of Rice RAD51A1 and RAD51A2

**Figure 1:**
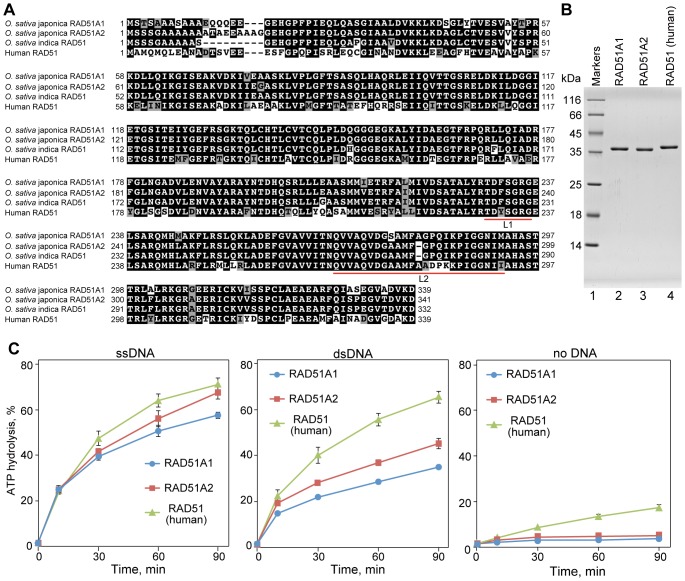
Purification of rice RAD51A1 and RAD51A2. (A) The amino acid sequences of rice RAD51A1 and RAD51A2 from japonica cultivar group, cv. Nipponbare, rice RAD51 from indica cultivar group, cv. Pusa Basmati 1, and human RAD51, aligned with the ClustalX software [50]. Black and gray boxes indicate identical and similar amino acid residues, respectively. The L1 and L2 loops, which are important for DNA binding, are represented by red lines. (B) Purified rice RAD51A1, RAD51A2, and human RAD51. Lane 1 indicates the molecular mass markers, and lanes 2, 3, and 4 represent rice RAD51A1 (0.5 µg), RAD51A2 (0.5 µg), and human RAD51 (0.5 µg), respectively. (C) The ATPase activities of *Oryza sativa* RAD51A1 and RAD51A2. The reactions were conducted with φX174 circular ssDNA (left panel), linearized φX174 dsDNA (center panel), or without DNA (right panel), in the presence of 5 µM ATP. Blue circles and red squares represent the experiments with RAD51A1 and RAD51A2, respectively. The averages of three independent experiments are shown with the SD values.

Two *RAD51* genes, *RAD51A1* and *RAD51A2*, have been identified in rice (japonica cultivar group). Only a single *RAD51* gene has been reported in an indica cultivar (Pusa Basmati 1) [36]. The amino acid sequence of the indica RAD51 is more similar to the japonica RAD51A2 (98%) than to RAD51A1 (93%) (Figure 1A).

RAD51A1 and RAD51A2 share 92% amino acid identity with each other, and both exhibit 69% amino acid identity with human RAD51, respectively (Figure 1A). To reveal the functional differences between RAD51A1 and RAD51A2, we expressed japonica rice RAD51A1 and RAD51A2 as recombinant proteins in *Escherichia coli* cells, and purified them by a three-step purification method (Figure 1B, lanes 2 and 3). We used *E. coli* cells lacking the *recA* gene, to eliminate the possibility of contamination with the RecA protein. The His_6_ tag was removed from the RAD51A1 and RAD51A2 portions by thrombin protease treatment, during the purification procedure.

The purified RAD51A1 and RAD51A2 hydrolyzed ATP in ssDNA- and dsDNA-dependent manners (Figure 1C). RAD51A2 exhibited higher ATP hydrolyzing activity than RAD51A1 in the presence of ssDNA or dsDNA (Figure 1C). The ATP hydrolyzing activities of RAD51A1 and RAD51A2 were lower than that of human RAD51 (Figure 1C). Under the experimental conditions used in this study, the kcat values of the ssDNA-dependent ATP hydrolyzing activities of RAD51A1, RAD51A2, and human RAD51 were about 0.06, 0.08, and 0.1 min^−1^, respectively.

### Rice RAD51A2 Exhibits Robust Homologous-pairing Activity

**Figure 2:**
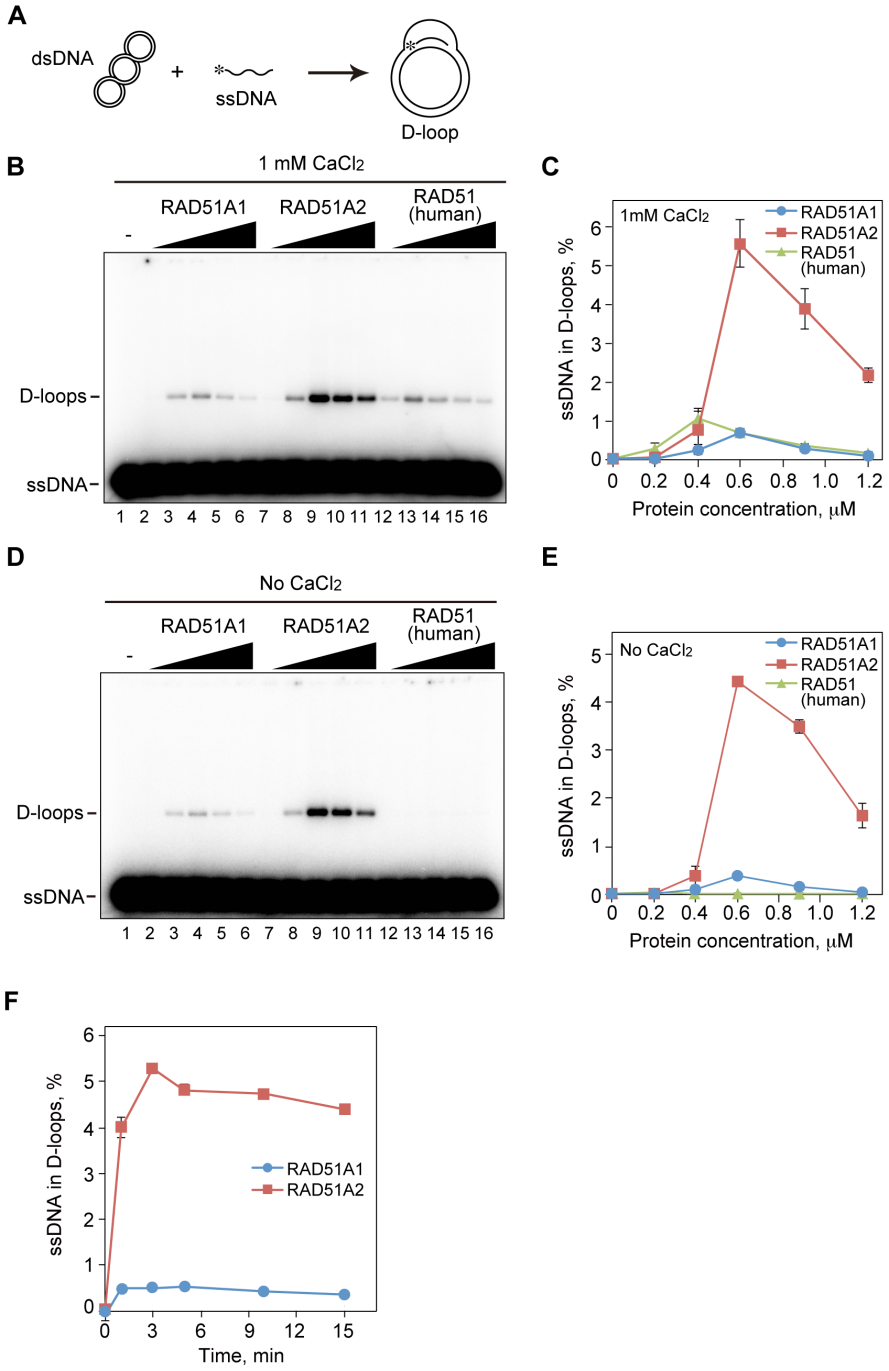
The homologous pairing activities of rice RAD51A1 and RAD51A2. (A) A schematic representation of the D-loop formation assay. Asterisks indicate the ^32^P-labeled end of the 50-mer ssDNA. (B) The D-loop formation assay in the presence of Ca^2+^(Protein titration experiments). The indicated amounts of rice RAD51A1, RAD51A2, or human RAD51 were incubated with the ^32^P-labeled 50-mer ssDNA, and the homologous-pairing reaction was initiated by the addition of superhelical dsDNA. Reactions were allowed to proceed for 5 min. Lane 1 indicates a negative control experiment without protein, and lanes 2–6, 7–11, and 12–16 represent the reactions conducted with RAD51A1, RAD51A2, and human RAD51, respectively. The protein concentrations were 0.2 µM (lanes 2, 7, and 12), 0.4 µM (lanes 3, 8, and 13), 0.6 µM (lanes 4, 9, and 14), 0.9 µM (lanes 5, 10, and 15), and 1.2 µM (lanes 6, 11, and 16). (C) Graphic representation of the experiments shown in panel B. The averages of three independent experiments are shown with the SD values. Blue circles, red squares, and green triangles represent the experiments with RAD51A1, RAD51A2, and human RAD51, respectively. (D) The D-loop formation assay without Ca^2+^(Protein titration experiments). The homologous pairing reactions were conducted without Ca^2+^, and were performed according to the same procedure as shown in panel B. (E) Graphic representation of the experiments shown in panel D. The averages of three independent experiments are shown with the SD values. Blue circles, red squares, and green triangles represent the experiments with RAD51A1, RAD51A2, and human RAD51, respectively. (F) Graphic representation of the D-loop formation assay (time course experiments). The homologous pairing reactions were conducted according to the same procedure as shown in panel B. Rice RAD51A1 (0.5 µM) or RAD51A2 (0.5 µM) was used in the time course experiments.

We next tested the homologous-pairing activities of RAD51A1 and RAD51A2. To this end, we performed D-loop formation assays. In these assays, D-loops were detected as a homologous pairing product, in which a ^32^P-labeled ssDNA fragment was hybridized to the complementary strand of superhelical dsDNA (Figure 2A). The homologous pairing mediated by the RecA-family proteins is an ATP- and Mg^2+^-dependent reaction [41], [42]. In addition, Ca^2+^ions reportedly stimulate the homologous pairing by human RAD51 [43]. Therefore, we conducted the homologous-pairing reactions in the presence of ATP, Mg^2+^, and Ca^2+^. We found that RAD51A2 robustly catalyzed homologous pairing, as compared to human RAD51 (Figure 2B and C). On the other hand, RAD51A1 exhibited the homologous-pairing activity, but it was only about 10% of the RAD51A2 activity under the conditions with ATP, Mg^2+^, and Ca^2+^(Figure 2B and C). Interestingly, RAD51A1 and RAD51A2 did not require Ca^2+^to catalyze homologous pairing, although the homologous-pairing activity of human RAD51 was hardly detected in the absence of Ca^2+^(Figure 2D and E). The homologous-pairing activity of human RAD51 was somewhat reduced under the 80 mM NaCl conditions employed for RAD51A1 and RAD51A2 (data not shown). RAD51A2 still exhibited about 12-fold higher homologous-pairing activity than RAD51A1 under the conditions without Ca^2+^ion (Figure 2D and E). In addition, time course experiments revealed that the D-loop formation by RAD51A2 became rapidly saturated within 3 min, and RAD51A2 possesses higher homologous-pairing activity than RAD51A1 (Figure 2F).

### The DNA-binding Activities of Rice RAD51A1 and RAD51A2

**Figure 3:**
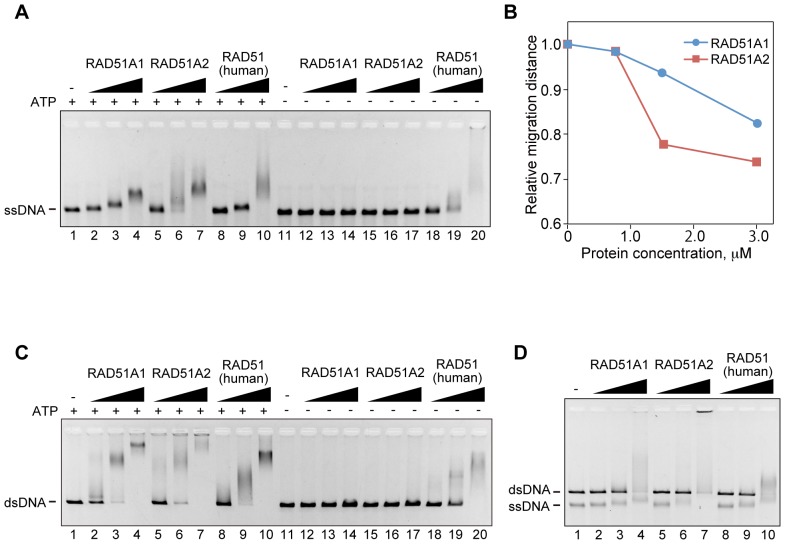
The DNA-binding activities of rice RAD51A1 and RAD51A2. Circular φX174 ssDNA (20 µM) (A) or linear φX174 dsDNA (20 µM) (C) was incubated with rice RAD51A1, RAD51A2, or human RAD51 at 37°C for 10 min. The samples were then separated by 0.8% agarose gel electrophoresis in TAE buffer, and were visualized by ethidium bromide staining. Lanes 1–10 and 11–20 represent the reactions conducted with and without ATP, respectively. Lanes 1 and 11 indicate negative control experiments without protein. Lanes 2–4 and 12–14 represent the experiments conducted with RAD51A1. Lanes 5–7 and 15–17 represent the experiments conducted with RAD51A2. Lanes 8–10 and 18–20 represent the experiments conducted with human RAD51. The protein concentrations were 0.75 µM (lanes 2, 5, 8, 12, 15, and 18), 1.5 µM (lanes 3, 6, 9, 13, 16, and 19) and 3 µM (lanes 4, 7, 10, 14, 17, and 20). (B) Graphic representation of the relative migration distances of the RAD51A1- and RAD51A2-ssDNA complexes. The migration distances relative to the free DNA are plotted against the protein concentrations. (D) Competitive ssDNA- and dsDNA-binding. Circular φX174 ssDNA (20 µM) and linear φX174 dsDNA (20 µM) were incubated with rice RAD51A1, RAD51A2, or human RAD51 at 37°C for 10 min, under the 120 mM NaCl conditions. The samples were then separated by 0.8% agarose gel electrophoresis in TAE buffer, and were visualized by ethidium bromide staining. Lane 1 indicates negative control experiments without protein. Lanes 2–4, 5–7, and 8–10 represent the experiments conducted with RAD51A1, RAD51A2, and human RAD51, respectively. The protein concentrations were 0.9 µM (lanes 2, 5, and 8), 1.8 µM (lanes 3, 6, and 9), and 3.6 µM (lanes 4, 7, and 10).

**Figure 4:**
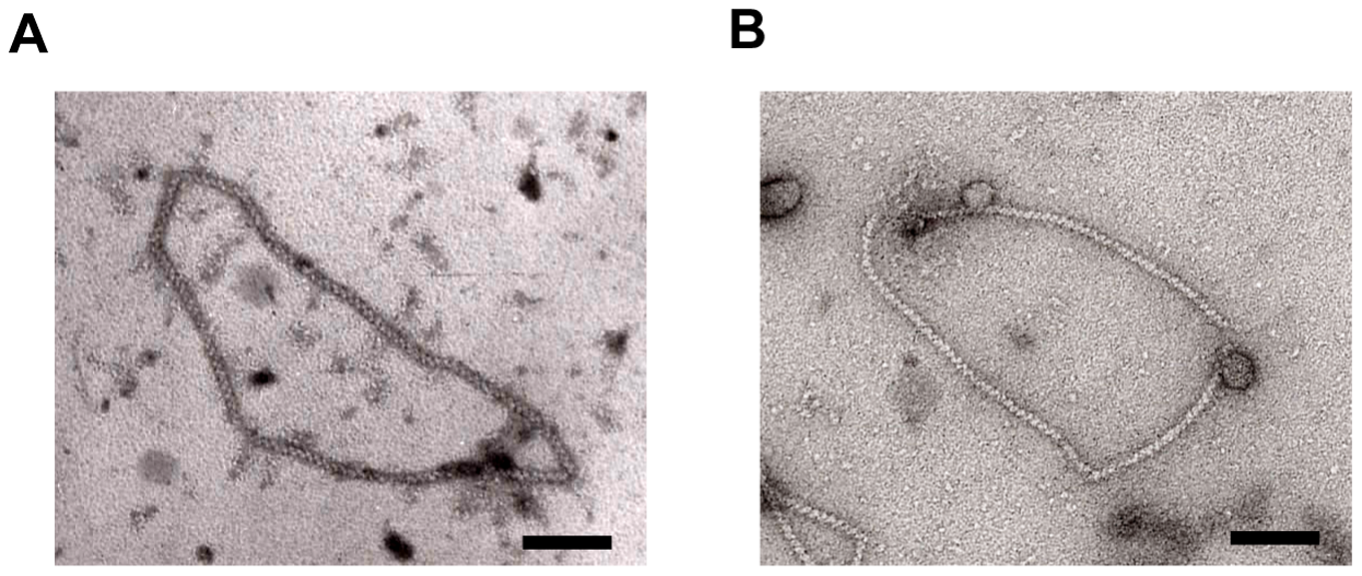
Electron microscopic images of RAD51A1 and RAD51A2 complexed with DNA. (A and B) Electron microscopic images of rice RAD51A1 (A) and RAD51A2 (B) filaments formed on the φX174 dsDNA in the presence of ATP. The average helical pitches of the RAD51A1 and RAD51A2 filaments were about 9.15 nm. The black bar denotes 100 nm.

We next tested the DNA-binding activities of RAD51A1 and RAD51A2. As shown in Figure 3A and C, both RAD51A1 and RAD51A2 bound to ssDNA and dsDNA, as well as human RAD51, in the presence of ATP (lanes 1–10). Interestingly, we found that RAD51A1 and RAD51A2 strictly required ATP for their ssDNA- and dsDNA-binding activities (Figure 3A and C, lanes 11–17), unlike human RAD51 (Figure 3A and C, lanes 18–20). Consistently, RAD51A1 and RAD51A2 formed helical nucleoprotein filaments with dsDNA in the presence of ATP (Figure 4A and B), but the filaments were not detected in the absence of ATP, due to their inability to bind DNA (Figure 3A and C). The average helical pitches of both the RAD51A1 and RAD51A2 filaments were about 9.15 nm. These results suggested that ATP modifies the polymerization states of RAD51A1 and RAD51A2, and enhances their DNA-binding activities.

Interestingly, the RAD51A2-ssDNA complexes migrated more slowly than the RAD51A1-ssDNA complexes (Figure 3A and B), suggesting that RAD51A2 may have higher ssDNA-binding activity than RAD51A1. In addition, competitive DNA binding assays revealed that RAD51A2, but not RAD51A1, significantly preferred to bind ssDNA (Figure 3D). The higher ssDNA-binding activity of RAD51A2 may be a major reason for its higher homologous-pairing activity (Figure 2).

### ATP-dependent Polymer Formation by Rice RAD51A1 and RAD51A2

**Figure 5:**
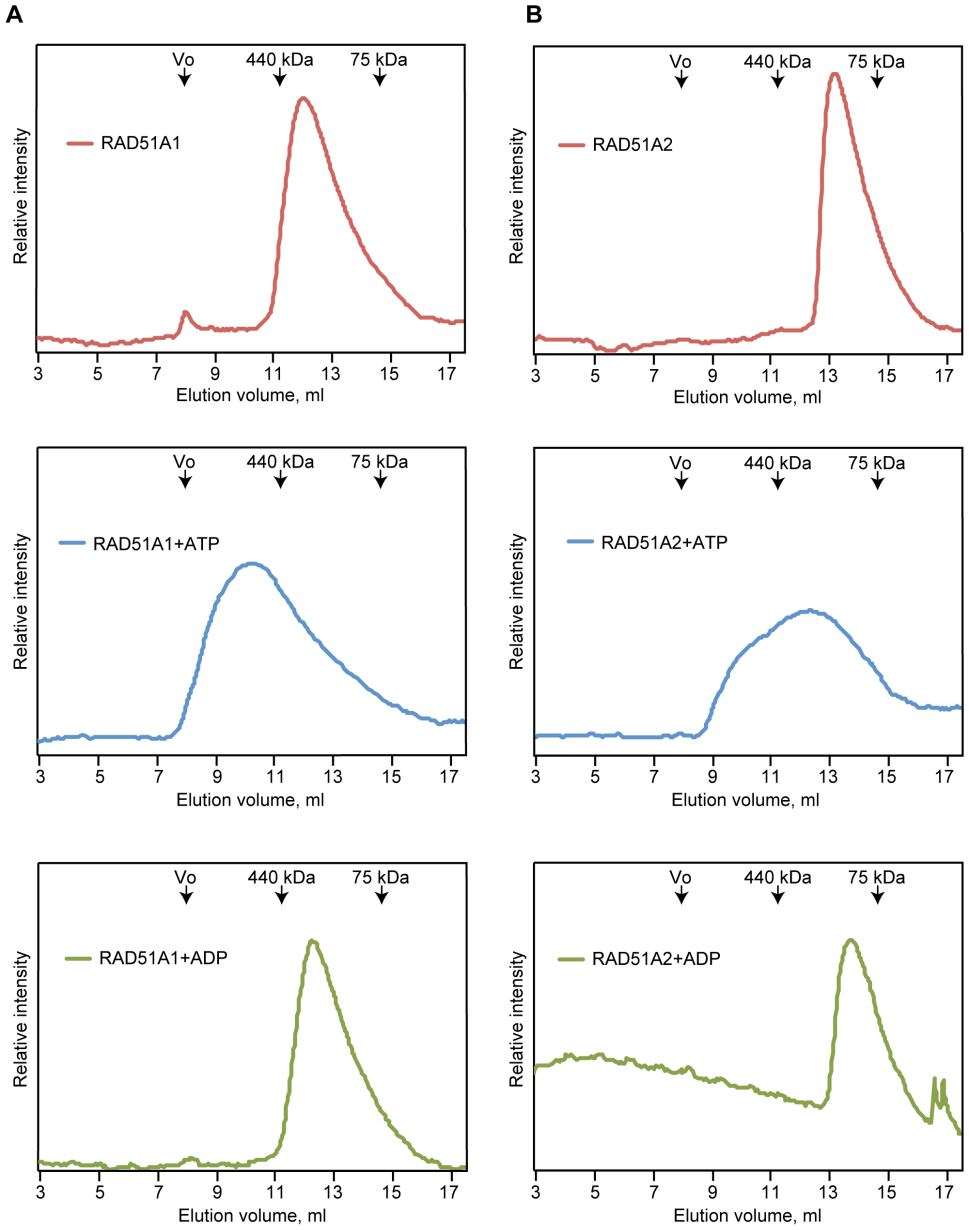
The polymerization activities of rice RAD51A1 and RAD51A2. A 70 µg portion of RAD51A1 (A) or RAD51A2 (B) was analyzed by Superdex 200 gel filtration chromatography. The top, middle, and bottom panels represent the experiments without ATP, with ATP, and with ADP, respectively.

To test whether ATP affects the polymerization states of RAD51A1 and RAD51A2, we tested the polymerization activities of RAD51A1 and RAD51A2 with or without ATP. To do so, we performed gel filtration chromatography. As shown in Figure 5 (top panels), RAD51A1 and RAD51A2 eluted at the apparent molecular sizes corresponding to 8-mer and 5-mer, respectively, without ATP. As expected, in the presence of ATP, the apparent molecular sizes of RAD51A1 and RAD51A2 drastically increased (Figure 5A and B, middle panels). These ATP-dependent changes in the RAD51A1 and RAD51A2 polymer formation were not induced by ADP (Figure 5A and B, bottom panels). Therefore, ATP actually enhances the oligomerization activities of RAD51A1 and RAD51A2. These ATP-dependent changes in the polymers formed by RAD51A1 and RAD51A2 may stimulate their DNA-binding activities during homologous pairing.

### Rice RAD51A1 and RAD51A2 may not form a co-filament

**Figure 6:**
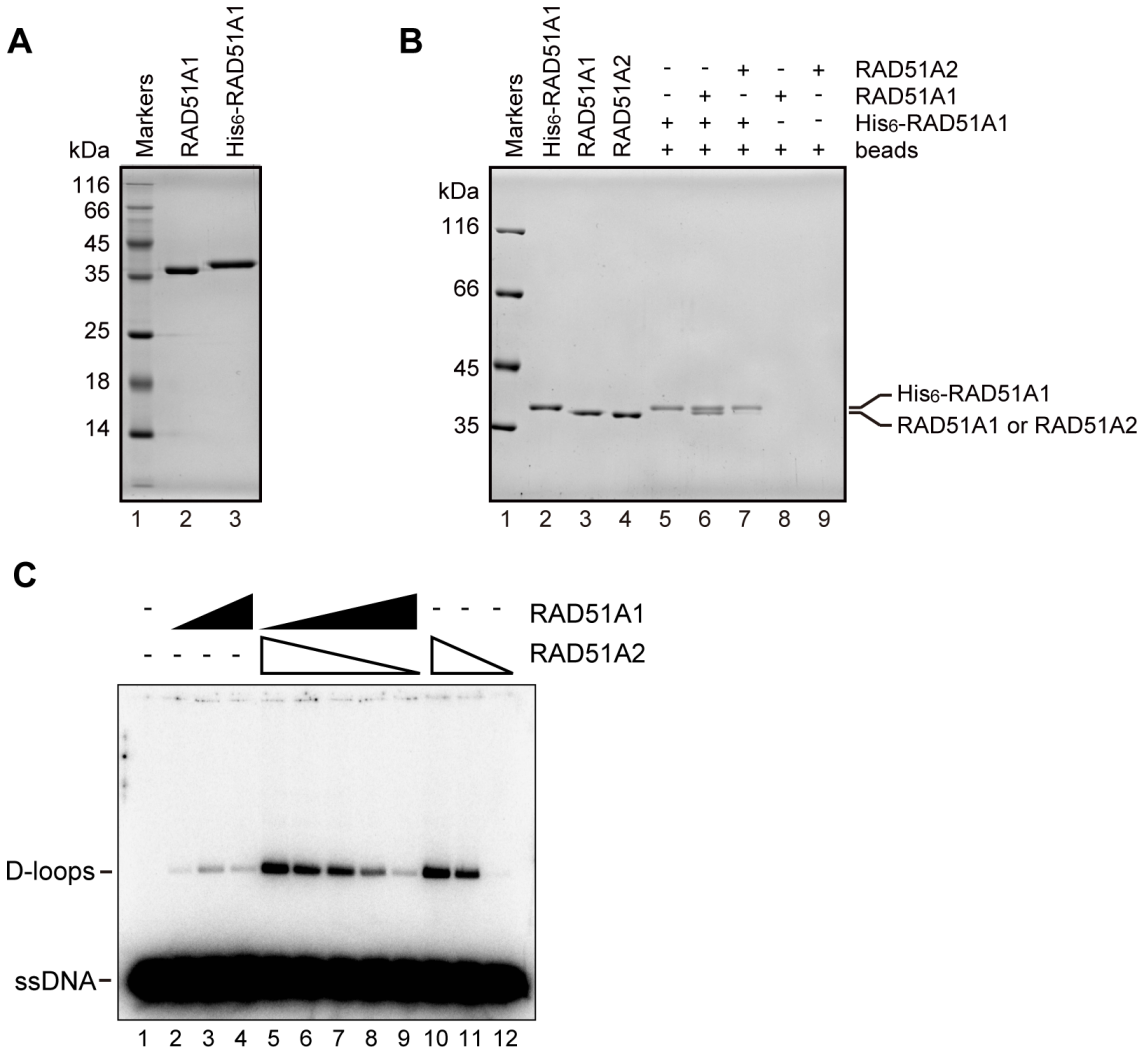
Interactions of rice RAD51A1 and RAD51A2. (A) Purified His_6_-tagged RAD51A1 protein. Lane 1 indicates the molecular mass markers, and lanes 2 and 3 represent the RAD51A1 and His_6_-tagged RAD51A1 proteins (0.5 µg). (B) The pull-down assay with Ni–NTA beads. Lane 1 represents molecular mass markers. Lanes 2, 3, and 4 show purified protein controls of His_6_-tagged RAD51A1, RAD51A1, and RAD51A2, respectively. Lane 5 indicates a negative control experiment without RAD51A1 and RAD51A2, in the presence of His_6_-tagged RAD51A1. Lanes 8 and 9 indicate negative control experiments with RAD51A1 and RAD51A2, respectively, in the absence of His_6_-tagged RAD51A1. Lanes 6 and 7 indicate experiments with RAD51A1 and RAD51A2, respectively, in the presence of His_6_-tagged RAD51A1. The proteins bound to His_6_-tagged RAD51A1 were pulled down by the Ni–NTA agarose beads. The samples were fractionated by 10% SDS–PAGE, and the protein bands were visualized by Coomassie Brilliant Blue staining. (C) The D-loop formation assay in the presence of Ca^2+^(Protein titration experiments). The indicated amounts of rice RAD51A1 and RAD51A2 were mixed and incubated with the ^32^P-labeled 50-mer ssDNA, and the homologous-pairing reaction was initiated by the addition of superhelical dsDNA. Reactions were allowed to proceed for 5 min. Lane 1 indicates a negative control experiment without protein, and lanes 2–4 and 10–12 indicate positive control experiments with RAD51A1 and RAD51A2, respectively. The protein concentrations were 0.2 µM (lanes 2 and 12), 0.4 µM (lanes 3 and 11), and 0.6 µM (lanes 4 and 10). Lanes 5–9 represent experiments with various amounts of RAD51A1 and RAD51A2. Lane 5: RAD51A1 (0 µM) and RAD51A2 (0.6 µM). Lane 6: RAD51A1 (0.2 µM) and RAD51A2 (0.4 µM). Lane 7: RAD51A1 (0.3 µM) and RAD51A2 (0.3 µM). Lane 8: RAD51A1 (0.4 µM) and RAD51A2 (0.2 µM). Lane 9: RAD51A1 (0.6 µM) and RAD51A2 (0 µM).

To test whether RAD51A1 and RAD51A2 form a co-filament, we performed the Ni-bead pull-down assay. In this assay, the His_6_-tagged RAD51A1 was purified (Figure 6A), and incubated with RAD51A1 or RAD51A2. The RAD51A1 or RAD51A2 bound to the His_6_-tagged RAD51A1 was detected. As shown in Figure 6B, RAD51A1 was efficiently pulled down by His_6_-tagged RAD51A1, but only a trace amount of RAD51A2 was detected. These results suggested that RAD51A1 efficiently binds to RAD51A1, but not to RAD51A2. Consistently, the homologous-pairing activities of RAD51A1 and RAD51A2 were not synergistically affected in the presence of various amounts of RAD51A1 and RAD51A2 (Figure 6C). Therefore, we concluded that rice RAD51A1 and RAD51A2 may not form a co-filament, and thus distinctly function in rice.

### Contribution of the L2 Loop Regions of Rice RAD51A1 and RAD51A2 to the Homologous-pairing Activity

Two flexible loops, L1 and L2, of human RAD51 are known to be important for its DNA-binding and homologous-pairing activities [44]. There are no differences in the amino acid sequences of the L1 loop regions between RAD51A1 and RAD51A2 (Figure 1A). In contrast, in the L2 loop region, the Ser276 residue of RAD51A1 is substituted with Ala279 in RAD51A2 (Figure 1A). In addition, one amino acid insertion, at the Ala280 residue of RAD51A1, is found in the L2 loop region (Figure 1A). These amino acid variations in the L2 loops region may be responsible for the differences in homologous pairing between RAD51A1 and RAD51A2.

**Figure 7:**
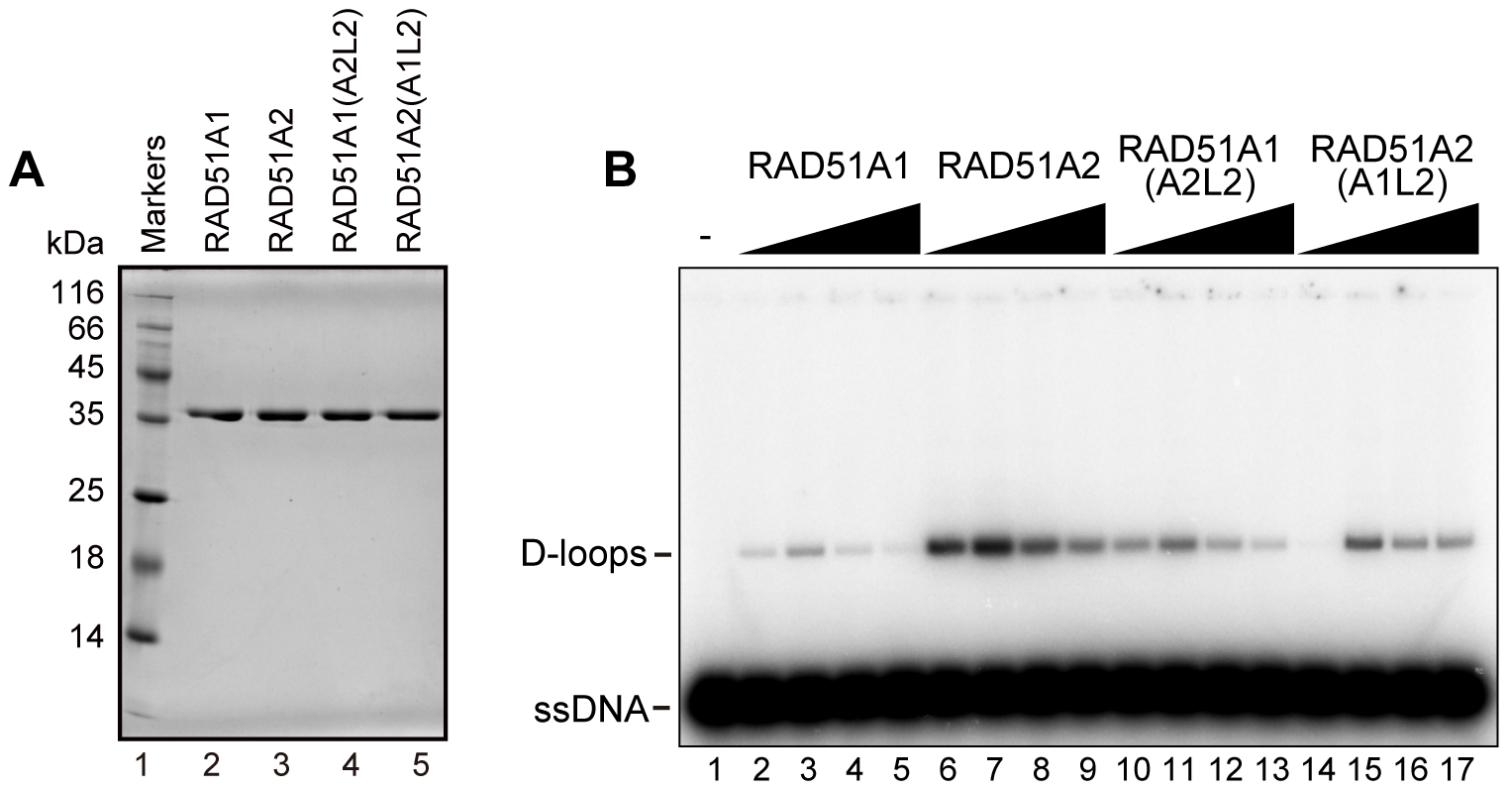
The homologous-pairing activities of the RAD51A1(A2L2) and RAD51A2(A1L2) mutants. (A) Purified RAD51A1(A2L2) and RAD51A2(A1L2) mutants. Lane 1 indicates the molecular mass markers, and lanes 2 and 3 represent RAD51A1 and RAD51A2, respectively. Lanes 4 and 5 represent RAD51A1(A2L2) and RAD51A2(A1L2). An aliquot (0.5 µg) of each protein was analyzed. (B) The D-loop formation assay (Protein titration experiments). The indicated amounts of rice RAD51A1, RAD51A2, RAD51A1(A2L2), or RAD51A2(A1L2) were incubated with the ^32^P-labeled 50-mer ssDNA, and the homologous-pairing reaction was initiated by the addition of superhelical dsDNA. Reactions were allowed to proceed for 5 min. Lane 1 indicates a negative control experiment without protein, and lanes 2–5, 6–9, 10–13, and 14–17 represent the reactions conducted with RAD51A1, RAD51A2, RAD51A1(A2L2), and RAD51A2(A1L2), respectively. The protein concentrations were 0.4 µM (lanes 2, 6, 10, and 14), 0.6 µM (lanes 3, 7, 11, and 15), 0.9 µM (lanes 4, 8, 12, and 16), and 1.2 µM (lanes 5, 9, 13, and 17).

Therefore, we constructed and purified the RAD51A1(A2L2) and RAD51A2(A1L2) mutants (Figure 7A), in which the L2 regions of RAD51A1 and RAD51A2 were replaced by the RAD51A2-L2 and RAD51A1-L2 regions, respectively. We then tested their homologous-pairing activities. Interestingly, the homologous-pairing activity of RAD51A1(A2L2) was clearly higher than that of the wild type RAD51A1 (Figure 7B, lanes 10–13). On the other hand, RAD51A2(A1L2) exhibited reduced homologous-pairing activity, as compared to the wild type RAD51A2 (Figure 7B, lanes 14–17). These results indicated that the L2 regions of RAD51A1 and RAD51A2 are at least partly responsible for their respective homologous-pairing activities.

## Discussion

Two RAD51 proteins, RAD51A1 and RAD51A2, have been identified in japonica cultivars of rice. A previous study revealed that the amount of *RAD51A2* mRNA, but not that of *RAD51A1* mRNA, is increased by ionizing irradiation, which induces DSBs [45]. The mRNA level of *RAD51A2* is higher than that of *RAD51A1* in embryogenic calli of rice, which exhibit higher recombination rates, as compared to other organs [46]. These facts suggested that RAD51A2 may be the major functional recombinase in the RAD51-mediated DSB repair and homologous recombination in rice. A *RAD51* gene has been identified in the rice indica-cultivar group [36]. The indica RAD51 shares 98% amino acid identity with the japonica RAD51A2, and 93% identity with the japonica RAD51A1. Since an additional locus for *RAD51*, which displays higher homology to *RAD51A1* than *RAD51A2*, has been found in the genome sequence of the indica rice cultivar Kasalath (J. Wu, personal communication), it is quite likely that two genes for RAD51 also exist in the indica cultivar group. We determined that RAD51A2 possesses robust homologous-pairing activity (Figure 2). RAD51A1 also catalyzes homologous pairing, but its activity is quite low, as compared to RAD51A2 (Figure 2). These biochemical findings are consistent with the idea that RAD51A2 is the major functional recombinase in rice.

However, the homologous-pairing activity of RAD51A1 may not be negligible, because it is comparable to that of human RAD51 (Figure 2). Therefore, RAD51A1 may not simply be a non-functional paralogue. Although the functional reasons for the diversity of RAD51A1 and RAD51A2 in rice have not been clarified yet, the biochemical distinctions and similarities of these two rice recombinases revealed in the present study may provide important insights into understanding their biological significance.

We found that RAD51A1 and RAD51A2 bind to ssDNA and dsDNA in ATP-dependent manners. These findings suggested that ATP may stabilize the protein-protein interactions within the RAD51A1 and RAD51A2 filaments. The crystal structures of the RadA protein, an archaeal RAD51 orthologue, and the RecA-DNA complexes revealed that the ATP molecule binds to the protein-protein interfaces within the filaments [47], [48]. The ATP bound to RAD51A1 and RAD51A2 may convert the RAD51 structures, enhancing the interactions between RAD51 monomers within the filament. Consistent with this idea, we found that ATP drastically changes the polymerization states of RAD51A1 and RAD51A2 (Figure 5). A similar ATP-dependent change in polymerization was also observed for human RAD51 (data not shown). In addition, the electrophoretic gel mobilities of the human RAD51-DNA complexes differ in the presence and absence of ATP, although human RAD51 binds DNA without ATP (Figure 3). The function of ATP in the formation of functional RAD51 filaments has been clarified with the rice RAD51A1 and RAD51A2 proteins.

In the present study, we detected significant differences in homologous pairing between RAD51A1 and RAD51A2, although they share high amino acid identity (92%). These biochemical differences may be a consequence of the amino acid differences in the DNA-binding regions. Bacterial RecA contains two flexible loops, L1 and L2, which are involved in DNA binding, and the corresponding L1 and L2 loops of human RAD51 are also important for its DNA-binding and recombination activities [44]. Although the amino acid sequences are the same in the L1 loop regions of RAD51A1 and RAD51A2, one amino acid difference (Ser276 in RAD51A1 and Ala279 in RAD51A2) and one amino acid insertion (Ala280) in RAD51A1 are found in the L2 loop region. In addition, it has been reported that the N-terminal fragment of human RAD51 (amino acids 1–114) directly binds to DNA [49]. Many amino acid differences exist in the corresponding N-terminal regions of RAD51A1 and RAD51A2. Therefore, these amino acid differences in the L2 loops and N-terminal regions may be responsible for the differences in homologous pairing between RAD51A1 and RAD51A2. In the present study, the D-loop formation assay with the L2-swapping mutants, RAD51A1(A2L2) and RAD51A2(A1L2), revealed that the L2 regions are actually responsible for their respective homologous-pairing activities. Intriguingly, the L2 loop sequence of japonica RAD51A2 is perfectly conserved with that of indica RAD51, which reportedly promotes homologous pairing [36], although the N-terminal sequence of indica RAD51 differs from those of both japonica RAD51A1 and RAD51A2. In fact, the L2-swapping mutations did not restore the homologous-pairing activities of wild type RAD51A1 and RAD51A2, indicating that the regions outside the L2 loop may also be important for the distinctive RAD51A1 and RAD51A2 activities. In addition, several amino acid differences also exist between RAD51A1 and RAD51A2 outside these DNA-binding regions. In a future study, it will be quite interesting to identify the amino acid residues responsible for the functional differences between RAD51A1 and RAD51A2.

## References

[1] Whitaker SJ (1992) DNA damage by drugs and radiation: what is important and how is it measured? Eur J Cancer 28: 273–276. 1567678 10.1016/0959-8049(92)90432-2

[2] Cox MM, Goodman MF, Kreuzer KN, Sherratt DJ, Sandler SJ, et al (2000) The importance of repairing stalled replication forks. Nature 404: 37–41. 10716434 10.1038/35003501

[3] Symington LS (2002) Role of RAD52 epistasis group genes in homologous recombination and double-strand break repair. Microbiol Mol Biol Rev 66: 630–670. 12456786 10.1128/MMBR.66.4.630-670.2002 PMC134659

[4] West SC (2003) Molecular views of recombination proteins and their control. Nat Rev Mol Cell Biol 4: 435–445. 12778123 10.1038/nrm1127

[5] San Filippo J, Sung P, Klein H (2008) Mechanism of eukaryotic homologous recombination. Annu Rev Biochem 77: 229–257. 18275380 10.1146/annurev.biochem.77.061306.125255

[6] Petronczki M, Siomos MF, Nasmyth K (2003) Un ménage à quatre: the molecular biology of chromosome segregation in meiosis. Cell 112: 423–440. 12600308 10.1016/s0092-8674(03)00083-7

[7] Neale MJ, Keeney S (2006) Clarifying the mechanics of DNA strand exchange in meiotic recombination. Nature 442: 153–158. 16838012 10.1038/nature04885 PMC5607947

[8] Shinohara A, Ogawa H, Ogawa T (1992) Rad51 protein involved in repair and recombination in S. cerevisiae is a RecA-like protein. Cell 69: 457–470. 1581961 10.1016/0092-8674(92)90447-k

[9] Aboussekhra A, Chanet R, Adjiri A, Fabre F (1992) Semidominant suppressors of Srs2 helicase mutations of *Saccharomyces cerevisiae* map in the *RAD51* gene, whose sequence predicts a protein with similarities to procaryotic RecA proteins. Mol Cell Biol 12: 3224–3234. 1620127 10.1128/mcb.12.7.3224-3234.1992 PMC364537

[10] Basile G, Aker M, Mortimer RK (1992) Nucleotide sequence and transcriptional regulation of the yeast recombinational repair gene *RAD51* . Mol Cell Biol 12: 3235–3246. 1620128 10.1128/mcb.12.7.3235-3246.1992 PMC364538

[11] Shinohara A, Ogawa H, Matsuda Y, Ushio N, Ikeo K, et al (1993) Cloning of human, mouse and fission yeast recombination genes homologous to *RAD51* and *recA* . Nat Genet 4: 239–243. 8358431 10.1038/ng0793-239

[12] Bishop DK, Park D, Xu L, Kleckner N (1992) *DMC1*: a meiosis-specific yeast homolog of E. coli *recA* required for recombination, synaptonemal complex formation, and cell cycle progression. Cell 69: 439–456. 1581960 10.1016/0092-8674(92)90446-j

[13] Habu T, Taki T, West A, Nishimune Y, Morita T (1996) The mouse and human homologs of *DMC1*, the yeast meiosis-specific homologous recombination gene, have a common unique form of exon-skipped transcript in meiosis. Nucleic Acids Res 24: 470–477. 8602360 10.1093/nar/24.3.470 PMC145652

[14] Sung P (1994) Catalysis of ATP-dependent homologous DNA pairing and strand exchange by yeast RAD51 protein. Science 265: 1241–1243. 8066464 10.1126/science.8066464

[15] Sung P, Robberson DL (1995) DNA strand exchange mediated by a RAD51-ssDNA nucleoprotein filament with polarity opposite to that of RecA. Cell 82: 453–461. 7634335 10.1016/0092-8674(95)90434-4

[16] Baumann P, Benson FE, West SC (1996) Human Rad51 protein promotes ATP-dependent homologous pairing and strand transfer reactions in vitro. Cell 87: 757–766. 8929543 10.1016/s0092-8674(00)81394-x

[17] Maeshima K, Morimatsu K, Horii T (1996) Purification and characterization of XRad51.1 protein, *Xenopus RAD51* homologue: recombinant XRad51.1 promotes strand exchange reaction. Genes Cells 1: 1057–1068. 9077454 10.1046/j.1365-2443.1996.d01-224.x

[18] Gupta RC, Bazemore LR, Golub EI, Radding CM (1997) Activities of human recombination protein Rad51. Proc Natl Acad Sci USA 94: 463–468. 9012806 10.1073/pnas.94.2.463 PMC19535

[19] Li Z, Golub EI, Gupta R, Radding CM (1997) Recombination activities of HsDmc1 protein, the meiotic human homolog of RecA protein. Proc Natl Acad Sci USA 94: 11221–11226. 9326590 10.1073/pnas.94.21.11221 PMC23422

[20] Hong EL, Shinohara A, Bishop DK (2001) Saccharomyces cerevisiae Dmc1 protein promotes renaturation of single-strand DNA (ssDNA) and assimilation of ssDNA into homologous super-coiled duplex DNA. J Biol Chem 276: 41906–41912. 11551925 10.1074/jbc.M105563200

[21] Kinebuchi T, Kagawa W, Enomoto R, Tanaka K, Miyagawa K, et al (2004) Structural basis for octameric ring formation and DNA interaction of the human homologous-pairing protein Dmc1. Mol Cell 14: 363–374. 15125839 10.1016/s1097-2765(04)00218-7

[22] Sehorn MG, Sigurdsson S, Bussen W, Unger VM, Sung P (2004) Human meiotic recombinase Dmc1 promotes ATP-dependent homologous DNA strand exchange. Nature 429: 433–437. 15164066 10.1038/nature02563

[23] Lim DS, Hasty P (1996) A mutation in mouse *rad51* results in an early embryonic lethal that is suppressed by a mutation in *p53* . Mol Cell Biol 16: 7133–7143. 8943369 10.1128/mcb.16.12.7133 PMC231717

[24] Tsuzuki T, Fujii Y, Sakumi K, Tominaga Y, Nakao K, et al (1996) Targeted disruption of the *Rad51* gene leads to lethality in embryonic mice. Proc Natl Acad Sci USA 93: 6236–6240. 8692798 10.1073/pnas.93.13.6236 PMC39005

[25] Sonoda E, Sasaki MS, Buerstedde JM, Bezzubova O, Shinohara A, et al (1998) Rad51-deficient vertebrate cells accumulate chromosomal breaks prior to cell death. EMBO J 17: 598–608. 9430650 10.1093/emboj/17.2.598 PMC1170409

[26] Stassen NY, Logsdon JM, Vora GJ, Offenberg HH, Palmer JD, et al (1997) Isolation and characterization of *rad51* orthologs from *Coprinus cinereus* and *Lycopersicon esculentum*, and phylogenetic analysis of eukaryotic *recA* homologs. Curr Genet 31: 144–157. 9021132 10.1007/s002940050189

[27] Doutriaux MP, Couteau F, Bergounioux C, White C (1998) Isolation and characterization of the *RAD51* and *DMC1* homologues from *Arabidopsis thaliana* . Mol Gen Genet 257: 283–291. 9520262 10.1007/s004380050649

[28] Franklin AE, McElver J, Sunjevaric I, Rothstein R, Bowen B, et al (1999) Three-dimensional microscopy of the Rad51 recombination protein during meiotic prophase. Plant Cell 11: 809–824. 10330467 10.1105/tpc.11.5.809 PMC144225

[29] Devisetty UK, Mayes K, Mayes S (2010) The *RAD51* and *DMC1* homoeologous genes of bread wheat: cloning, molecular characterization and expression analysis. BMC Res Notes 3: 245. 20920212 10.1186/1756-0500-3-245 PMC2962619

[30] Markmann-Mulisch U, Hadi MZ, Koepchen K, Alonso JC, Russo VEA, et al (2002) The organization of *Physcomitrella patens RAD51* genes is unique among eukaryotic organisms. Proc Natl Acad Sci USA 99: 2959–2964. 11880641 10.1073/pnas.032668199 PMC122455

[31] Franklin AE, Golubovskaya IN, Bass HW, Cande WZ (2003) Improper chromosome synapsis is associated with elongated RAD51 structures in the maize *desynaptic2* mutant. Chromosoma 112: 17–25. 12811575 10.1007/s00412-003-0242-8 PMC13613107

[32] Pawlowski WP, Golubovskaya IN, Cande WZ (2003) Altered nuclear distribution of recombination protein RAD51 in maize mutants suggests the involvement of RAD51 in meiotic homology recognition. Plant Cell 15: 1807–1816. 12897254 10.1105/tpc.012898 PMC167171

[33] Li W, Chen C, Markmann MU, Timofejeva L, Schmelzer E, et al (2004) The *Arabidopsis AtRAD51* gene is dispensable for vegetative development but required for meiosis. Proc Natl Acad Sci USA 101: 10596–10601. 15249667 10.1073/pnas.0404110101 PMC489980

[34] Markmann-Mulisch U, Wendeler E, Zobell O, Schween G, Steinbiss HH, et al (2007) Differential requirements for RAD51 in *Physcomitrella patens* and *Arabidopsis thaliana* development and DNA damage repair. Plant Cell 19: 3080–3089. 17921313 10.1105/tpc.107.054049 PMC2174717

[35] Ayora S, Piruat JI, Luna R, Reiss B, Russo VEA, et al (2002) Characterization of two highly similar Rad51 homologs of *Physcomitrella patens* . J Mol Biol 316: 35–49. 11829501 10.1006/jmbi.2001.5336

[36] Rajanikant C, Melzer M, Rao BJ, Sainis JK (2008) Homologous recombination properties of OsRad51, a recombinase from rice. Plant Mol Biol 68: 479–491. 18695945 10.1007/s11103-008-9385-6

[37] Sakane I, Kamataki C, Takizawa Y, Nakashima M, Toki S, et al (2008) Filament formation and robust strand exchange activities of the rice DMC1A and DMC1B proteins. Nucleic Acids Res 36: 4266–4276. 18583359 10.1093/nar/gkn405 PMC2490746

[38] Kurumizaka H, Aihara H, Kagawa W, Shibata T, Yokoyama S (1999) Human Rad51 amino acid residues required for Rad52 binding. J Mol Biol 291: 537–548. 10448035 10.1006/jmbi.1999.2950

[39] Ishida T, Takizawa Y, Sakane I, Kurumizaka H (2008) The Lys313 residue of the human Rad51 protein negatively regulates the strand-exchange activity. Genes Cells 13: 91–103. 18173750 10.1111/j.1365-2443.2007.01143.x

[40] Kagawa W, Kurumizaka H, Ikawa S, Yokoyama S, Shibata T (2001) Homologous pairing promoted by the human Rad52 protein. J Biol Chem 276: 35201–35208. 11454867 10.1074/jbc.M104938200

[41] Shibata T, DasGupta C, Cunningham RP, Radding CM (1979) Purified *Escherichia coli recA* protein catalyzes homologous pairing of superhelical DNA and single-stranded fragments. Proc Natl Acad Sci U S A 76: 1638–1642. 156361 10.1073/pnas.76.4.1638 PMC383445

[42] McEntee K, Weinstock GM, Lehman IR (1979) Initiation of general recombination catalyzed in vitro by the recA protein of *Escherichia coli* . Proc Natl Acad Sci U S A 76: 2615–2619. 379861 10.1073/pnas.76.6.2615 PMC383658

[43] Bugreev DV, Mazin A (2004) Ca^2+^activates human homologous recombination protein Rad51 by modulating its ATPase activity. Proc Natl Acad Sci USA 101: 9988–9993. 15226506 10.1073/pnas.0402105101 PMC454202

[44] Matsuo Y, Sakane I, Takizawa Y, Takahashi M, Kurumizaka H (2006) Roles of the human Rad51 L1 and L2 loops in DNA binding. FEBS J 273: 3148–3159. 16780572 10.1111/j.1742-4658.2006.05323.x

[45] Endo M, Nakayama S, Umeda-Hara C, Ohtsuki N, Saika H, et al (2012) CDKB2 is involved in mitosis and DNA damage response in rice. Plant J 69: 967–977. 22092531 10.1111/j.1365-313X.2011.04847.x PMC3440594

[46] Yang Z, Tang L, Li M, Chen L, Xu J, et al (2010) Monitoring homologous recombination in rice (*Oryza sativa* L.). Mutat Res 691: 55–63. 20670635 10.1016/j.mrfmmm.2010.07.005

[47] Wu Y, He Y, Moya IA, Qian X, Luo Y (2004) Crystal structure of archaeal recombinase RADA: a snapshot of its extended conformation. Mol Cell 15: 423–435. 15304222 10.1016/j.molcel.2004.07.014

[48] Chen Z, Yang H, Pavletich NP (2008) Mechanism of homologous recombination from the RecA-ssDNA/dsDNA structures. Nature 453: 489–494. 18497818 10.1038/nature06971

[49] Aihara H, Ito Y, Kurumizaka H, Yokoyama S, Shibata T (1999) The N-terminal domain of the human Rad51 protein binds DNA: structure and a DNA binding surface as revealed by NMR. J Mol Biol 290: 495–504. 10390347 10.1006/jmbi.1999.2904

[50] Thompson JD, Gibson TJ, Plewniak F, Jeanmougin F, Higgins DG (1997) The CLUSTAL_X windows interface: flexible strategies for multiple sequence alignment aided by quality analysis tools. Nucleic Acids Res 25: 4876–4882. 9396791 10.1093/nar/25.24.4876 PMC147148

